# Role of platelet chemokines, PF-4 and CTAP-III, in cancer biology

**DOI:** 10.1186/1756-8722-6-42

**Published:** 2013-06-24

**Authors:** Katerina Pilatova, Kristina Greplova, Regina Demlova, Beatrix Bencsikova, Giannoula Lakka Klement, Lenka Zdrazilova-Dubska

**Affiliations:** 1 Department of Laboratory Medicine, Masaryk Memorial Cancer Institute, Zluty kopec 7, Brno 656 53, Czech Republic; 2 Department of Clinical Evaluation, Masaryk Memorial Cancer Institute, Zluty kopec 7, Brno 656 53, Czech Republic; 3 Clinic of Comprehensive Cancer Care, Masaryk Memorial Cancer Institute, Zluty kopec 7, Brno 656 53, Czech Republic; 4 Center of Cancer Systems Biology, Steward St. Elizabeth’s Medical Center Pediatric Hematology Oncology, Tufts University School of Medicine, Boston, MA, USA; 5 Department of Pharmacology, Medical Faculty, Masaryk University, Kamenice 5, Brno 625 00, Czech Republic; 6 Regional Centre for Applied Molecular Oncology, Masaryk Memorial Cancer Institute, Zluty kopec 7, Brno 656 53, Czech Republic

**Keywords:** Platelet, PF-4, CTAP-III, Cancer, Angiogenesis, IL-8, Inflammation

## Abstract

With the recent addition of anti-angiogenic agents to cancer treatment, the angiogenesis regulators in platelets are gaining importance. Platelet factor 4 (PF-4/CXCL4) and Connective tissue activating peptide III (CTAP-III) are two platelet-associated chemokines that modulate tumor angiogenesis, inflammation within the tumor microenvironment, and in turn tumor growth. Here, we review the role of PF-4 and CTAP-III in the regulation of tumor angiogenesis; the results of clinical trial using recombinant PF-4 (rPF-4); and the use of PF-4 and CTAP-III as cancer biomarkers.

## Introduction

Angiogenesis regulators are sequestered in platelets [1]. Platelet factor 4 and Connective tissue activating peptide III constitute two major platelet CXC chemokines [2]. CXC chemokines have four highly conserved cystein residues with the two N-terminal cysteines separated by one amino acid residue. CXC chemokines are subdivided into two classes, ELR^+^ and ELR^-^, based on the presence or absence of specific amino acid sequence (ELR, Glu-Leu-Arg) [3]. CXC chemokines are generally implicated in inflammatory angiogenesis, and the ELR motif plays an important role in whether the specific CXC chemokine promotes or inhibits angiogenesis. ELR-containing chemokines, such as CTAP-III, are pro-angiogenic, while ELR-lacking chemokines, such as PF-4 are angiostatic [3-5].

The following is a review of the role of PF-4 and CTAP-III in inhibition and regulation of tumor angiogenesis, respectively; results from rPF-4 clinical trial; and PF-4 and CTAP-III as cancer biomarkers.

### PF-4 physiology and function

PF-4 is heparin-binding polypeptide belonging to the ELR^-^ CXC chemokine family. PF-4 is a tetrameric molecule, with each subunit consisting of 70 amino acid residues with molecular weight 7.8 kDa [6]. The human gene encoding PF-4 maps to 4q12-21 [7]. PF-4 is synthesized almost exclusively by megakaryocytes and sequestered in platelet α-granules [8]. Upon activation, platelets release tetrameric PF-4 bound to two molecules of chondroitin sulphate proteoglycan, which is displaced by heparin binding [9]. Physiological platelet levels of PF-4 have been reported about 7–22 ng PF-4/10^6^ cells [10,11] which is about 150 μg/ml. Plasma levels of PF-4 are strongly dependent on platelet activation *in vitro*[10-14]; e.g. the levels in plasma supplemented by inhibitors of platelet function are as low as 1.8 ± 1 ng/ml [13], while levels of PF-4 measured in citrated tubes can be as high as 150–360 ng/ml (10,14). Similarly, high serum levels (about 5 μg/ml) correlate with platelet counts [13].

PF-4 shows both procoagulant and anticoagulant activity. It can prevent heparin binding to antithrombin leading to inhibition of heparin-dependent thrombin inactivation [15]. On the other hand, the inhibition of factor XII (intrinsic or contact activation pathway) and that of vitamin K-dependent coagulation factors can lead to PF-4-mediated anticoagulant activity [16,17]. PF-4 further inhibits coagulation by generation of activated protein C by thrombomodulin binding [18]. In addition to its function in thrombosis and hemostasis, PF-4 plays an important role in wound healing, atherosclerosis and tumor biology mainly through its ability to regulate angiogenesis and function of different immune cell types. Furthermore, PF-4 [19-23] as well as CTAP-III [19] have been shown to inhibit megakaryocytopoiesis. PF-4 also inhibits proliferation of erythroid and granulocyte/macrophage colonies [20,24] and CD34+ progenitors via IL-8 interaction [25].

### CTAP-III physiology and function

**Figure 1:**
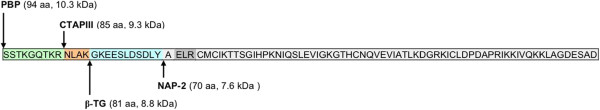
**Amino acid sequences of β-thromboglobulin-like proteins **[36]**.**

CTAP III is a major platelet ELR^+^ CXC chemokine with molecular weight 9.3 kDa [26]. It is produced not only by megakaryocytes but also by monocytes, lymphocytes and neutrophils [27,28]. CTAP-III, along with β-thromboglobulin (β-TG), platelet basic protein (PBP) and neutrophil-activating peptide 2 (NAP-2, CXCL7) belongs to β-thromboglobulin-like proteins (Figure 1). CTAP-III is converted from a precursor PBP, the major megakaryocytes variant, during megakaryocyte maturation and platelet formation [29]. After its release from platelet α-granules, CTAP-III can be proteolytically cleaved to β-TG [26,30] and/or NAP-2 [31,32] with chemotactic activity [33]. The exact mechanism of the cleavage regulation is unknown. Although CTAP-III, β-TG and NAP-2 are all NH_2_-terminal truncated variants of the PBP precursor, each possesses a very distinct biological function. NAP-2 acts like a typical CXC chemokine while longer forms (PBP and CTAP-IlI) has no that activity [33]. The PBP gene is localized to 4q12-q13 in the vicinity PF-4 gene [7]. The manner in which CTAP-III stimulates connective tissue cells [26,34,35] and its immunoregulatory activity as a precursor of NAP-2 [31] are summarized in Table 1.

**Table 1:** CTAP III biological functions

**Regulation of hematopoiesis**	Inhibition of megakaryocytopoiesis [19]
**Immunoregulatory activity**	Histamine release by basophils [37]
	Precursor of NAP-2 [31]
**Angiogenesis**	Chemotaxis of EC *in vitro*[5]
**Connective tissue cells metabolism**	Mitogenesis [34]
	Glycolysis [34]
	Hyaluronic acid and GAGs synthesis [22,34]
	Prostaglandin E_2_ secretion [22]
	Plasminogen activator synthesis [35]
**Others**	Stimulation of glucose transport [38]
	Transcellular mediator of the cellular sphingomyelin import [39]
	Heparanase activity [40]

### Platelet regulation of tumor angiogenesis and tumor growth

Angiogenesis in adults play an important role in wound healing, female reproductive cycle but also in pathologic processes, such as diabetic retinopathy, cancer and other inflammatory disorders [41]. The process of angiogenesis is regulated by balance of positive and negative regulators. Platelet α-granules contain both types of angiogenesis regulators and consequently, platelets are involved in tumor angiogenesis [42-44]. Positive regulators of angiogenesis include platelet-derived growth factor (PDGF), vascular endothelial growth factor (VEGF), fibroblast growth factor-2 (FGF-2) etc.; and angiogenesis inhibitors include PF-4, endostatin, thrombospondin-1 etc. [45]. Italiano et al. (2008) reported that angiogenic and anti-angiogenic factors are stored in distinct sets of α-granules and their release is regulated by selective activation of different thrombin receptors [46].

**Figure 2:**
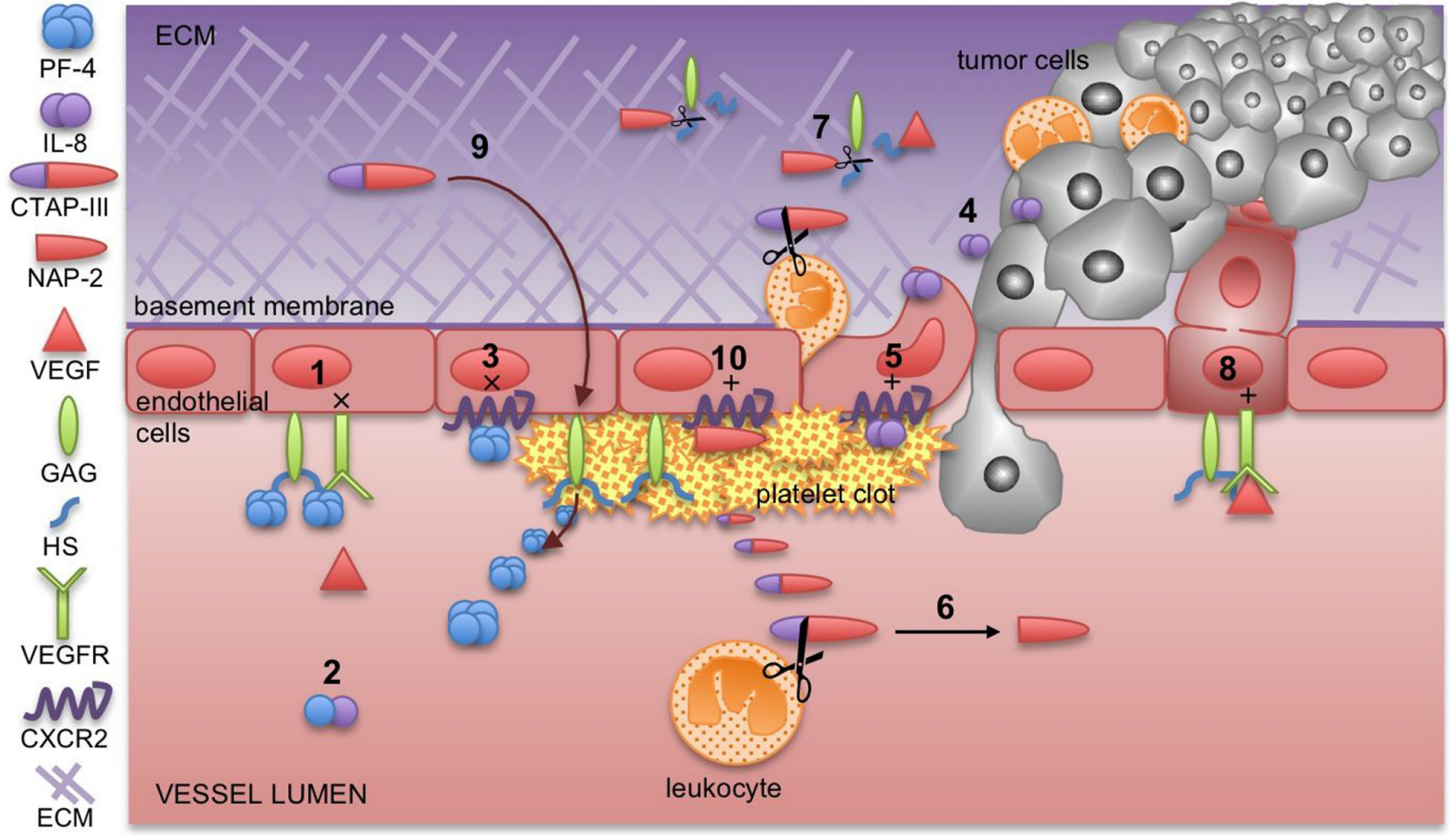
**PF-4 and CTAP-III/NAP-2 connection and their role in tumor angiogenesis and progression.** The interaction of various components of the wound or tumor stroma depends on the presence or absence of different tissue proteases and on the reciprocal interaction of the various cells. PF-4 inhibits angiogenesis by (**1**) competitive inhibition of pro-angiogenic growth factors binding to HS sites in the tissues, where HS serve as co-receptor of growth factor receptors, such as VEGFR2 [47,48]; (**2**) by binding of IL-8 to form PF-4/IL-8 heterodimers [49] (**3**) and by antagonising of CXCR2, IL-8 receptor, which is involved in regulation inflammation and angiogenesis [50]. (**4**) IL-8, released by tumor as well as stroma and endothelial cells, induces chemotaxis of inflammatory cells [51] and angiogenesis [52] (**5**) by signaling through CXCR2 receptor [52]. The CXCL7/CTAP-III acts in a number of different ways: (**6**) CTAP-III is cleaved by leukocyte proteases to NAP-2 [31,32]; (**7**) NAP-2 splits HS from glycosaminosulfates (GAG) in the stroma, leading to interruption of extracellular matrix (ECM) protein-protein interactions and release of heparan sulphate-bound growth factors, such as VEGF [40]. (**8**) The remodeled ECM at the side of inflammation and angiogenesis enables the interaction of the released growth factors (e.g. VEGF) with their respective receptors and leads to modulation of angiogenesis and regulation of tumor spreading [53]; (**9**) CTAP-III stimulates further GAG synthesis [54] on the surface of endothelial cell injury leading to increased PF-4 production and localization of heparin binding angiogenesis regulators. (10) NAP-2 regulates activity and expression of CXCR2 [51,55].

The role of PF-4 and CTAP-III in regulation of angiogenesis and within the tumor microenvironment is described in following paragraphs and the main features are summarized in Figure 2.

### PF-4, as an angiogenesis inhibitor

PF-4 inhibits migration and proliferation [47,56,57] of endothelial cells (EC) and angiogenesis both *in vitro*[58] and *in vivo*[57,59] via several mechanisms. Firstly, PF-4 binds positive angiogenesis regulators such as VEGF, bFGF and thus prevents their receptor binding and bFGF dimerization [47,48,60,61]. More specifically, PF-4 also impedes growth factor binding to its proteoglycan receptors by competition for heparin and heparan sulphate (HS) sites or by displacement growth factor from these sites [47,48]. Lasagni et al. (2003) has described PF-4 receptor CXCR3B, a variant of chemokine receptor CXCR3, which is expressed on microvascular endothelium and activated T-lymphocytes [62]. PF-4/CXCR3B signaling plays a role in transduction of apoptotic signal and inhibition of proliferation in endothelium [62,63]. A further mechanism of inhibition of endothelial cell growth and proliferation has recently been reported by Woller et al. (2008) showing that reactive oxygen radicals released from PF-4-activated monocytes are responsible for the induction of apoptosis in EC [64]. PF-4 also prevents entry of EC into S phase and DNA synthesis [56]. A PF-4 variant (CXCL4L1/PF-4var) differing from its native compound in three amino acids at the peptide carboxy-terminal part is even more potent angiogenesis inhibitor, and its role in cancer biology is reviewed by Vandercappellen et al. 2011 [65].

PF-4 also exerts its anti-angiogenic activity via inhibition of pro-angiogenic cytokine IL-8. IL-8, also a CXC chemokine family member, has been shown to enhance endothelial cell survival, proliferation, and production of matrix metalloproteinases which further stimulates tumor angiogenesis, and consequently tumor growth and metastasis [52]. IL-8 exhibits its signaling via CXCR2 receptor which is under normal conditions antagonized by PF-4 [50]. Moreover, PF-4/IL-8 heterodimers have stronger anti-proliferative activity on endothelial cells than PF-4 alone [49]. Inhibitory effect of recombinant PF-4 on tumor growth and metastasis [66,67] is most likely consequence of tumor angiogenesis inhibition, because PF-4 does not inhibit proliferation of tumor cells *in vitro*[66]. Gene therapy by PF-4 gene transfer has also shown anticancer effect *in vivo*[68,69].

### Regulation of tumor angiogenesis and tumor growth: connection with tumor microenvironment and the immune system

Inflammation is the primary and likely the most important host protective reaction to tissue and cellular damage. However, many pathological processes including cancer may recruit inflammatory response. Immune cells are endowed with a dual role: as a defense mechanism, or as a supporter of tumor growth particularly by stimulation of EC and tumor neovascularization in a process referred to as “inflammatory angiogenesis”. It is thought to be due changes within a tumor microenvironment that stimulates immune regulators to release cytokines and growth factors that lead to promotion of tissue remodeling, angiogenesis and tumor growth [70].

Although PF-4 belongs to the chemokine family it doesn’t show significant chemotactic activity for neutrophils [36,70-72]. Yet, PF-4 is involved in regulation of other cell types through other mechanisms which involve complex spectrum of functions on immune cells as summarized in Table 2. Reports on the ability of PF-4 to stimulate innate immune response predominate suggesting that rather than using inflammation to stimulate tumor growth, PF-4 stimulates immune cancer surveillance and tumor inhibition.

**Table 2:** PF-4 biological functions

**Procoagulant activity**	Inhibition of heparin-dependent thrombin inactivation [15]
**Anticoagulant activity**	Inhibition of factor XII [16] and vitamin K dependent coagulation factors [17]
	Generation of activated protein C [18]
**Regulation of hematopoiesis**	Inhibition of megakaryocytopoiesis [19-24]
	Survival of hematopoietic and progenitor cells [73]
	Inhibition of BFU-E, CFU-GM and CD34+ progenitors proliferation [24,25]
**Immunoregulatory activity**	Stimulation of neutrophil adhesion and secondary granule exocytosis [36,71,72,74]
	Stimulation of monocyte adhesion and activation [64,75,76]
	Induction of monocyte differentiation into macrophage [77] and APC [78]
	Stimulation of eosinophil adhesion [79]
	Stimulation of histamine release by basophils [80]
	Activation of NK cells and IL-8 release [81]
	Inhibition of T-cell activation and proliferation [82]
**Anti-angiogenic activity**	Growth factors (VEGF, bFGF) binding [47,48,60]
	Competition with growth factors (VEGF, bFGF) for glycosaminoglycan (GAGs) binding [47,48]
	Prevention of EC entry into S phase and inhibition of DNA synthesis [56]
	Monocyte ROS mediated cytotoxicity for EC [64]

### CTAP-III, as an angiogenesis modulator and stimulator of inflammation

There is not much information about the role of CTAP-III in angiogenesis but expression studies suggest that CTAP-III plays an important role in tumor growth and progression. CTAP-III has been reported to mediate chemotaxis of EC *in vitro* and stimulate angiogenesis *in vivo*[5]. Of these various cleavage products of PBP, only CTAP III possesses heparanase activity rendering it a very distinct role in modulating tumor progression [53].

NAP-2/CXCL7, CTAP-III cleavage product, has been shown to stimulate angiogenesis *in vivo*[83]. NAP-2 also stimulates neutrophil degranulation leading to increased vascular permeability [84]. Together, CTAP-III and NAP-2 collaborate in degrading heparin and heparan sulphate [40], important components of extracellular matrix and anchoring proteins for many heparin-binding regulators of angiogenesis. As the surface of inflammatory and endothelial cells in the tumor microenvironment expresses increased amounts of HS, local blood coagulation, fibrin deposition, cell adhesion and tumor growth are facilitated. Tang et al. 2008 reported that CXCL7 transfected breast cells acquired invasive properties and demonstrated elevated heparanase activity, which caused remodeling of extracellular matrix and facilitate cancer metastasis [53].

NAP-2 is formed through further cleavage of PBP and CTAP-III in the presence of leukocyte proteases [31,32,51]. While its precursors do not show pro-inflammatory activity, NAP-2 stimulates both chemotaxis and neutrophil degranulation through chemokine receptors CXCR-1 and CXCR-2 [33,51]. The amino-terminal residues of NAP-2 extended variants probably mask ELR motif, a crucial neutrophil receptor binding domain, leading to predominantly inhibitory chemokine activity [85]. However, it has been shown that continuous accumulation of NAP-2, as a product of PBP and CTAP-III proteolysis, results in anti-inflammatory activity by desensitization of neutrophils through down-regulation of chemokine receptors, especially CXCR-2. This finding suggests that NAP-2 has dual function and that interaction of the various PBP cleavage products produces a very finely tuned system.

### PF-4 in clinical trials

Clinical trials testing recombinant PF-4 have been completed in metastatic colon cancer [86], AIDS-related Kaposi’s sarcoma [87,88], metastatic melanoma, renal cell carcinoma [89] and high-grade glioma [90]. The phase I trial in patients with metastatic colorectal cancer evaluated 9 patients who had failed 5-FU treatment. Subjects received rPF-4 at doses ranging from 0.3 to 3.0 mg/kg via 30-minute infusion, three additional patients were treated with the 3 mg/kg dose using a 6-hour infusion. Of the 11 evaluable patients, rPF-4 was well tolerated at the doses and schedules tested, but no clinical responses to treatment rPF-4 were observed. Similar results were observed in phase I study of recombinant platelet factor 4 in patients with metastatic melanoma and renal cell carcinoma. Three dosage groups with 3 patients at each level of 0.3, 1.0 and 3.0 mg/kg were evaluated. Recombinant PF-4 was given as a 6-hour infusion on days 1, 8–10 and 15–19 and could be given in two 5 day courses on days 29–33 and 43–47. All patients completed the initial 9 doses and 4 completed the 19 additional doses. There was no hematopoietic, hepatic, renal or coagulation toxicity, and most of the symptoms were attributed to the underlying disease. No dose response was recorded. Six patients progressed and two were stable during the 7 week study period. The authors concluded that rPF-4 had no biological activity at the doses and schedules used.

These perceived failures may be due to the fact that PF-4, similarly to other biologic response modifiers, is a sensitizer to cytotoxic chemotherapy rather than a cytotoxic agent and its effect may not be detected in monotherapy setting. Furthermore, establishing a maximally tolerated dose of rPF-4 in phase I may be inappropriate. Most biologic response modifiers, rPF-4 included, have U-shaped response curves and maximum effect is achieved at mid-range. High doses lead to toxicities caused by undesirable (and unnecessary) off-target effects. Thus, the goal when using biologic response modifiers such as rPF-4 should be the determination of a biologically effective dose. However, establishing the optimal dose may be very difficult in absence of validated surrogate markers for its biological activity. At least for now, the choice of phase I trial designs and appropriate end points may need to be guided by the mechanism of action of the agent like rPF-4. Currently no phase II trials with rPF-4 have been continued.

### PF-4 and CTAP-III as biomarkers of tumor growth

PF-4 and CTAP-III can be used as biomarkers of tumor growth [1,10,91,92]. Yee et al. (2009) identified higher serum levels of CTAP-III in pulmonary venous than in arterial blood using mass spectrometry (MS) and immunoassay [93]. While the levels of CTAP-III decreased after curative surgical resection, the elevated levels did not decrease in patients with residual disease after resection. Elevated blood levels of CTAP-III were detectable for up to 29 months before clinical diagnosis of lung cancer [93]. Increased levels of plasma CTAP-III were also detected using MS and ELISA in patients with lung cancer [94].

Grisaru et al. (2000) studied tissue expression of CTAP-III in cervical cancer specimens using immunostaining [95]. In normal epithelium CTAP-III was distributed in all of the epithelial layers, except in the highly active and proliferating basal cells. Cells of invasive cervical carcinoma did not stain for CTAP-III, and the presence of CTAP-III was limited to endothelial cells of capillary blood vessels. Moreover, CTAP-III staining pattern correlated positively with the degree of epithelial cell differentiation and with the stage of CIN [95] suggesting the role of CTAP-III in tumor progression and angiogenesis.

As it appears, PF-4 is concentrated in platelets and little is detected in plasma [91,92]. Platelet levels of PF-4 as determined by surface-enhanced laser desorption/ionization time-of-flight MS (SELDI TOF MS) are up-regulated following implantation of human tumor xenografts in mice but fall in tumor progression. In contrast, the levels of PF-4 in plasma remained unchanged [1,91,92]. We had proposed that elevated platelet levels in tumor-bearing mice present feedback loop mechanism in response to the induction of pro-angiogenic factors by the growing tumor [91]. Study in patients with early colorectal cancer showed statistically significant increase in PF-4 in platelets coincident with a rise in pro-angiogenic factors, such as VEGF and PDGF, compared to healthy controls, while changes in plasma levels of PF-4 remained insignificant [92]. It is likely that PF-4 rather than being released from platelets in circulation, binds locally to the HS at sites of platelet adhesion.

## Conclusions

Until recently, PF-4 has been studied in the past mainly in the setting of heparin-induced thrombocytopenia. The clinical translation of its biological effects in suppression of tumor growth, prevention of atherosclerotic plague, endometriosis, chronic inflammation and other angiogenesis-dependent diseases may have been hindered by a lack of understanding of its biological effects and mechanism of action. We have summarized emerging data on role of PF-4 and CTAP-III in regulation of tumor growth. It appears that the role of these two chemokines in modulation of tumor dynamics cannot be separated from the role of platelets and inflammation within the tumor microenvironment. While much of the biology of platelet-associated PF-4 and CTAP-III is likely to be harnessed with therapeutic intent only in the future, an obvious immediate clinical application may be to use them as biomarkers of cancer presence and/or therapeutic response. The more conventional biological samples such as serum or plasma have certainly not lead to emergence of any reliable biomarker. However, since angiogenesis regulators are sequestered in platelets, measurement of these chemokines in platelets may give a much better reflection of the actual angiogenic process. Finally, PF-4 is a locally acting protein whose role is to modulate the stroma of the wound or the tumor and its systemic administration may not ensure its bioavailability within the respective microenvironment. Perhaps the delivery of a recombinant PF-4 via platelets as its natural vehicle may provide a more biologically relevant treatment modality and improve its therapeutic potential.

## Competing interests

The authors declare that they have no competing interests.

## Authors’ contributions

KP drafted chapters "introduction", "PF-4 physiology and function CTAP-III physiology and function" and "Platelet regulation of tumor angiogenesis and tumor growth"; prepared tables and figures; formatted references. KG drafted chapters "PF-4 and CTAP-III as biomarkers of tumor growth". RD drafted chapter "PF-4 in clinical trials". BB drafted chapter "PF-4, as an angiogenesis inhibitor"; contributed to table 2 preparation. GLK drafted chapter "CTAP-III, as an angiogenesis modulator and stimulator of inflammation"; revised manuscript. LZD came up with an idea and design of the manuscript; drafted chapter "Regulation of tumor angiogenesis and tumor growth connection with tumor microenvironment and the immune system" and "conclusions"; finalized the manuscript. All authors read and approved the final manuscript.
